# Profiles of sleep hygiene, insomnia, sleep locus of control, and psychological well-being among nursing students: a cluster analysis

**DOI:** 10.3389/fpsyg.2026.1920757

**Published:** 2026-09-03

**Authors:** Ramya Kundayi Ravi, Daljit Kaur, Shaimaa Hashem Elsalous, Nilda Ensomo Ogsimer, Gifty Thomas, Lakshmi Ramamoorthy, Jaison Jacob, Priya Baby

**Affiliations:** 1 RAK College of Nursing, RAK Medical and Health Sciences University, Ras al-Khaimah, United Arab Emirates; 2 SPHE College of Nursing, Mohali, India; 3 College of Nursing, JIPMER, Puducherry, India; 4 Faculty, College of Nursing, AIIMS Bhubaneswar, Bhubaneswar, India; 5 College of Nursing, NIMHANS, Bengaluru, India

**Keywords:** cluster analysis, insomnia, locus of control, mental health, nursing students, psychological well-being, sleep hygiene, India

## Abstract

**Introduction:**

Psychological well-being is crucial for nursing students, who often face academic and clinical stressors that disrupt their sleep patterns. Insomnia severity, sleep hygiene, and sleep locus of control are key sleep-related factors that potentially influence mental health. However, the combined effects of these factors on psychological well-being remain underexplored, particularly among Indian nursing students.

**Methods:**

A cross-sectional analytical study was conducted among 418 nursing students from two nursing colleges in Northwestern India. Data were collected using validated instruments measuring insomnia severity (Athens Insomnia Scale), sleep hygiene (Sleep Hygiene Index), sleep locus of control (Sleep Locus of Control Scale), and psychological well-being (WHO-5 Well-Being Index). Statistical analyses included descriptive statistics, Spearman’s correlation, two-stage cluster analysis (hierarchical and k-means), and multiple linear regression, with statistical significance set at *p* < 0.05.

**Results:**

Three distinct sleep profiles were identified: insomnia-prone (21.3%), intermediate (51.4%), and adaptive (27.3%). The Insomnia-Prone cluster exhibited the highest insomnia severity, poorest sleep hygiene, least adaptive sleep locus of control beliefs, and lowest psychological well-being. The Adaptive cluster showed the most favorable sleep characteristics and highest well-being. Regression analysis revealed that insomnia severity (*β* = −0.31, *p* < 0.001), poor sleep hygiene (*β* = −0.13, *p* = 0.004), and chance-oriented sleep locus of control (*β* = −0.12, *p* = 0.04) significantly predicted lower psychological well-being, collectively explaining 20% of the variance in the data.

**Conclusion:**

The three distinct sleep profiles identified in this study highlight the differential vulnerability of psychological well-being across the student population. Sleep-related behavioral and cognitive factors are associated with the psychological well-being of nursing students. Addressing maladaptive sleep patterns through tailored interventions could support the psychological well-being of nursing students.

## Introduction

Sleep is a fundamental biological process that plays a vital role in cognitive functioning, emotional regulation, and physical health (Tang et al., 2023). A recent metaanalysis estimated a pooled prevalence of insomnia at 25.7% among Indians (Datta et al., 2025). University students in India are also shown to have high burden of sleep problems with 66% reporting poor sleep quality (Kumari et al., 2020). Among university students, health care students including nursing students are highly vulnerable to poor sleep and well-being (Alwhaibi and Al Aloola, 2023). The demanding nature of nursing education, encompassing rigorous coursework and clinical training, often disrupts sleep patterns and contributes to mental health issues (Alwhaibi and Al Aloola, 2023; Ravi et al., 2025; Mithurja et al., 2026). Consequently, understanding the interplay between sleep-related factors and psychological well-being in nursing students is essential for developing effective interventions to support their academic success and long-term health (Nielson et al., 2023).

Sleep-related key constructs, such as sleep hygiene and sleep locus of control, shape sleep health. Sleep hygiene refers to a set of behavioral and environmental practices conducive to sleeping well regularly. These practices include maintaining a consistent sleep schedule, minimizing noise and light in the sleeping environment, and avoiding stimulants or electronics before going to bed (Kaur, 2024). Poor sleep hygiene is a significant predictor of diminished sleep quality and is linked to various negative psychosocial outcomes (Malik et al., 2024). Sleep Locus of Control (SLOC) is a cognitive construct that evaluates the degree to which an individual attributes their sleep experiences to internal causes (such as their own behaviors and efforts) or external causes (such as chance or powerful others; Vieira et al., 2023). Individuals with an “internal” locus of control believe they can influence their sleep outcomes through their actions, whereas those with a “chance” or “external” locus of control often feel powerless regarding their sleep quality. A chance-oriented SLOC has been associated with more severe insomnia symptoms and lower self-efficacy in managing sleep disturbances (Vieira et al., 2023).

Insomnia is a common sleep disorder characterized by persistent difficulty in falling asleep, staying asleep, or experiencing non-restorative sleep despite having an adequate opportunity for rest. In student populations, insomnia is frequently associated with stressors such as academic workload and problematic mobile phone use (PMPU; Albikawi, 2023; Jose et al., 2024). It serves as a major indicator of sleep dysfunction and is strongly correlated with adverse mental health conditions, including depression, anxiety, and reduced satisfaction with life (Samrkhanova et al., 2023; Jose et al., 2024).

Psychological Well-being encompasses an individual’s emotional, social, and psychological functioning. It is conceptualized through indicators such as mood stability, positive mental health, and the absence of distress (Samrkhanova et al., 2023; Kaur, 2024). Research indicates that sleep quality and hygiene are factors that are significantly associated with psychological well-being (Amalia and Asih, 2024; Malik et al., 2024).

Nursing students represent a unique cohort that balances the demands of higher education with the responsibilities of clinical practice. Current evidence highlights the substantial burden of sleep problems among Indian students (da Silva et al., 2019; Ravi et al., 2025). While national estimates specifically for nursing students are limited, related cohorts provide a concerning baseline. For instance, a multisite study of Indian yoga and naturopathy medical students found that 59.8% experienced poor sleep quality and 24.7% practiced poor sleep hygiene (Silwal et al., 2025).

Despite growing evidence on sleep health among university students, the role of Sleep Locus of Control (SLOC) remains underexplored in India. Most existing studies on nursing students have primarily focused on sleep quality and sleep hygiene (Vieira et al., 2023). Sleep Locus of Control might be especially relevant for Indian nursing students because they often face situations outside their full control. Most of the nursing students stay in hostels, have to perform shift duties as part of clinical requirements and have substantial academic demands; all of which may leave students with limited personal control over their choice of sleep times (Brandwein, 2011; Jose et al., 2024; Kaur, 2024). Additionally, very few studies have analyzed insomnia severity, sleep hygiene, and sleep locus of control together within a single framework. Furthermore, much of the available evidence originates from international settings, such as Brazil, Saudi Arabia, and China, limiting the generalizability of the findings to the sociocultural and academic contexts of Indian nursing students (da Silva et al., 2019; Wu X. et al., 2022; Albikawi, 2023).

Methodologically, previous studies have largely explored the association between sleep related factors and psychological well-being, with limited exploration about underlying patterns (Mishra et al., 2022; Kaur, 2024; Kaur et al., 2024). Person-centered analytical methods, such as cluster analysis can pinpoint naturally occurring subgroups of students who share similar patterns of insomnia severity, sleep hygiene habits, and sleep control beliefs. This type of profiling supports the creation of targeted, context-specific interventions rather than using generalized approaches to improve sleep health (Silwal et al., 2025). Building on this rationale, the present study was undertaken with the following objectives.

### Objectives

- To assess the levels of insomnia, sleep hygiene, sleep locus of control, and psychological well-being among nursing students.
- To identify distinct clusters of nursing students based on sleep-related factors, including insomnia severity, sleep hygiene, and sleep locus of control.
- To explore differences in psychological well-being across the identified sleep-related clusters among nursing students.
- To determine the extent to which sleep-related factors are associated with psychological well-being among nursing students.

### Hypotheses

Based on the existing literature the following hypotheses were formulated

1. Distinct sleep related clusters, characterized by different sleep profiles would emerge among the nursing students.
2. Insomnia prone cluster would have lowest pyschological well-being among the other identified clusters.
3. Insomnia severity, poor sleep hygiene and external locus of control would predict lower psychological well-being.

## Methodology

### Design and setting

A quantitative approach with a cross-sectional analytical design was adopted for the study and reported in accordance with the STROBE Guidelines. The study was conducted in two nursing colleges conveniently selected from the Northwestern region of India. These institutions offer undergraduate and postgraduate nursing programs. The findings of the study should hence be considered specifc to the context and should not be generalized to all Indian nursing students.

### Population and sample

The target population comprised nursing students enrolled in GNM, B. Sc. Nursing, and M. Sc. Nursing programs. The accessible population included nursing students available during the period of data collection at the selected nursing institution. The study included nursing students who were enrolled in the selected institutes during the academic year 2025–2026 aged 18 years and above, and willing to participate in the study. Students who were absent during data collection, unwilling to participate, or medically diagnosed with any mental health illness were excluded from the study.

### Sample size calculation

The sample size requirements for multiple regression analysis were estimated using G*Power 3.1. Assuming a medium effect size (f^2^ = 0.15), *α* = 0.05, power = 0.80, and three predictors, a minimum sample of 77 participants was required. The final sample of 418 nursing students substantially exceeded this requirement. In addition, the sample was considered adequate for cluster analysis, as the clustering procedure included only four variables (insomnia, sleep hygiene, internal sleep locus of control, and chance sleep locus of control), resulting in a participant-to-variable ratio greater than 100:1. Methodological literature suggests that larger samples contribute to the stability and interpretability of cluster solutions, particularly when the number of clustering variables is relatively small and clusters are adequately represented. Furthermore, all identified clusters contained substantial proportions of the sample (21.3–51.4%), supporting the reliability of the cluster solution (Siddiqui, 2013; Dalmaijer et al., 2022).

### Measures

A structured questionnaire comprising four sections was used to collect data (Supplementary file 1). The English versions of the questionnaire was used as Eglish is the medium of instruction of the students enrolled in the study and they were comfortable in completing the questionnaire in English.

Section 1: A structured demographic questionnaire with five sections was used to collect participants’ baseline characteristics and selected study variables to collect information regarding age, gender, year of study, and history of chronic illness.

Section 2: Insomnia severity was assessed using the Athens Insomnia Scale (AIS), a self-administered instrument developed to evaluate sleep difficulty based on the International Classification of Diseases (ICD-10) insomnia criteria (Soldatos et al., 2000). The scale consists of eight items assessing sleep induction, awakenings during the night, final awakening, total sleep duration, sleep quality, daytime well-being, functional capacity, and daytime sleepiness. Each item is rated on a 4-point Likert scale ranging from 0 (no problem) to 3 (very serious problem), yielding a total score ranging from 0 to 32, with higher scores indicating greater severity of insomnia. The AIS has demonstrated good psychometric properties, with Cronbach’s alpha values reported between 0.83 and 0.89 (Soldatos et al., 2000). The reliability of the Athens Insomnia Scale in the present study was 0.80.

Section 3: Sleep hygiene behaviors were measured using the Sleep Hygiene Index (SHI), a 13-item self-report instrument designed to assess behaviors and practices that may interfere with sleep quality (SHI; Mastin et al., 2006). Each item is rated on a 5-point Likert scale ranging from 0 (never) to 4 (always). The total score ranges from 0 to 52, with higher scores reflecting poorer sleep hygiene practices and greater engagement in behaviors detrimental to sleep. Lower scores indicate healthier sleep hygiene. The SHI has demonstrated acceptable internal consistency, with a reported Cronbach’s alpha of 0.66, and good test–retest reliability (Mastin et al., 2006). The reliability of the Sleep Hygiene Index in the present study was found to be 0.88.

Beliefs regarding personal control over sleep were assessed using the Sleep Locus of Control Scale (SLOC; Shahid et al., 2012). The SLOC is an 8-item questionnaire designed to evaluate the extent to which individuals attribute their sleep experiences to internal or external factors. Responses were rated on a 6-point Likert scale ranging from 1 (strongly disagree) to 6 (strongly agree). Items 3, 4, and 6 were reverse scored. The total score ranges from 8 to 48, with higher scores indicating a stronger internal locus of control regarding sleep, whereas lower scores reflect greater attribution to external or chance factors. The scale demonstrated moderate internal consistency, with Cronbach’s alpha values ranging from 0.47–0.73 (Shahid et al., 2012). The reliability of the Sleep Locus of Control Scale in the present study was found to be 0.82. This tool has not been validated in Indian population and it was found to be appropriate for use in Indian culture. However, as formal validation has not been undertaken in Indian settings, this should be considered while inetrepreting results.

Psychological well-being was measured using the WHO-5 Well-Being Index, a brief self-reported instrument developed by the World Health Organization to assess subjective psychological well-being (Topp et al., 2015; Cosma et al., 2022). The scale consists of five positively worded items rated on a 6-point Likert scale from 0 (at no time) to 5 (all of the time), based on experiences over the previous 2 weeks. The total raw scores range from 0 to 25 and are multiplied by four to obtain a final score ranging from 0 to 100, with higher scores indicating better psychological well-being. Scores below 50 suggest poor well-being and possible depression. The WHO-5 has demonstrated strong reliability and validity across different populations, with Cronbach’s alpha values ranging from 0.82 to 0.90, indicating good internal consistency (Topp et al., 2015). The reliability of the well-being scale in the present study was found to be 0.85.

### Data collection

Data were collected during April 2026 using a structured questionnaire developed in Google Forms. The survey was administered in classroom settings during students’ free periods to minimize disruption to academic activities. Prior to participation, all students were provided with detailed information regarding the purpose and nature of the study, and written informed consent was obtained electronically. Only those who provided consent were directed to complete the survey. Students completed the survey in the class room under the supervision of a member of the research team. No discussion with peers or use of external resources was allowed during the completion of questionnaire. Students were permitted to raise clarifications regarding the items without influencing their response. Participation was voluntary, and anonymity and confidentiality were maintained throughout the study. No personal identifiers, such as names, email addresses, university identification numbers, or IP addresses, were collected. All responses were recorded anonymously and used solely for research purposes. A total of 464 students were approached for the survey of which 418 students returned the completed form, yeileding a response rate of 90.1%. The Google Form’s inbuilt response restruction setting was used to prevent same repsonent submitting multiple responses. To ensure completeness of data all items were made mandatory.

### Statistical analysis

Data were analyzed using the Statistical Package for the Social Sciences (SPSS) version 28. Descriptive statistics, including frequency, percentage, mean, and standard deviation, were used to summarize the demographic characteristics and study variables. Correlation analysis was performed to examine the relationships between psychological well-being, insomnia, sleep hygiene, and sleep locus of control. Prior to clustering, these variables were standardized (z-scores) to ensure equal weighting in the clustering procedure. Cluster analysis was conducted using a two-stage approach involving hierarchical clustering with Euclidean distance and Ward’s method, followed by k-means clustering to identify distinct sleep-related profiles based on insomnia, sleep hygiene, and sleep locus-of-control variables. Psychological well-being was not included in the cluster formation process and was subsequently compared across identified clusters. The optimal number of clusters was determined by examining the agglomeration schedule and dendrogram from the hierarchical clustering procedure. This cluster solution was subsequently refined using K-means clustering. All clustering variables were standardized to z-scores before the analysis.

Multiple linear regression analysis was performed to determine whether insomnia, sleep hygiene, and sleep locus of control predicted psychological well-being. Prior to analysis, the assumptions of normality and homogeneity of variance (homoscedasticity) were assessed, and minor deviations from normality and unequal variance of residuals were observed. The statistical significance was set at *p* < 0.05.

### Ethical considerations

Ethical approval for this study was obtained from the Institutional Ethics Committee of SPHE College of Nursing (IEC/ SPHE/2026/208). Administrative permission to conduct the study was obtained from the respective institutional authorities prior to the data collection. Written informed consent was obtained from all participants prior to participation. This study was conducted in accordance with the ethical principles of the Declaration of Helsinki.

## Results

Table 1 presents the characteristics of the 418 nursing students who participated in the study, with a mean age of 21.09 ± 2.70 years. The majority of participants were female (88.8%) and reported no history of chronic illness (97.8%). More than half of them were enrolled in the GNM program (53.8%), followed by B. Sc. program (41.6%).

**Table 1:** Sociodemographic and clinical characteristics of the participants.

Variable	Frequency	Percentage(%)
Age; Mean (SD)	21.09 (2.70)	
Gender		
Male	47	11.2%
Female	371	88.8%
Program of study		
GNM	225	53.8%
B. Sc nursing	174	41.6%
M. Sc Nursing	19	4.5%
Presence of chronic illness		
Yes	9	2.2%
No	409	97.8%

Table 2 presents the descriptive statistics of the study variables. Nursing students reported moderate to good psychological well-being, with a mean score of 72.76 ± 19.48 and a median score of 19.0 (IQR: 16.0–21.0). The median insomnia score was 3.0 (IQR: 1.0–7.0), indicating generally low levels of insomnia among participants. The median sleep locus of control score was 31.0 (IQR: 29.0–33.0), with higher internal locus of control scores (median = 23.0, IQR: 20.0–26.0) and lower chance locus of control scores (median = 8.0, IQR: 6.0–10.0). The median sleep hygiene score was 13.0 (IQR: 7.25–19.0), suggesting relatively favorable sleep hygiene practices among the students.

**Table 2:** Descriptive statistics of psychological well-being, insomnia, sleep locus of control, and sleep hygiene.

Variables	Total score possible	Minimum	Maximum	Mean ± SD	Median (IQR)
Well-being	0–100	0	100	72.76 ± 19.48	19.0 (16.0–21.0)
Insomnia	0–32	0	24	4.60 ± 4.20	3.0 (1.0–7.0)
Sleep LOC	8–48	20	42	31.28 ± 3.19	31 (29–33)
Internal LOC	5–30	9	30	23.24 ± 4.20	23.0 (20.0–26.0)
Chance LOC	3–18	3	18	8.04 ± 3.28	8.0 (6.0–10.0)
Sleep hygiene	0–52	0	39	13.56 ± 8.34	13.0 (7.25–19.0)

LOC, Locus of Control.

Table 3 presents the correlations between psychological well-being, insomnia, sleep locus of control, and sleep hygiene. As the data were not normally distributed, Spearman’s rank-order correlation was used for all the analyses. Psychological well-being showed a moderate negative correlation with insomnia (*ρ* = −0.42, *p* < 0.01), indicating that higher insomnia severity was associated with lower well-being. Low negative correlations were observed between psychological well-being and both chance sleep locus of control scores (ρ = −0.20, *p* < 0.01) and sleep hygiene scores (ρ = −0.28, *p* < 0.01), indicating that students who more strongly attributed sleep to chance factors and those who reported poorer sleep hygiene practices had lower levels of well-being. A low positive correlation was found between psychological well-being and internal sleep locus of control score (ρ = 0.24, *p* < 0.01), indicating that students with greater perceived control over sleep had higher levels of well-being. A strong negative correlation was observed between the internal and chance sleep locus of control (ρ = −0.66, *p* < 0.01).

**Table 3:** Correlation between psychological well-being, insomnia, sleep locus of control, and sleep hygiene.

Variables	Psychological well-being	Insomnia	Internal LOC	Chance LOC	Sleep hygiene
Well-being	1				
Insomnia	−0.42** p < 0.001	1			
Internal LOC	0.24** p < 0.001	−0.12* p = 0.01	1		
Chance LOC	−0.20** p < 0.001	0.003, *p* = 0.95	−0.66** p < 0.001	1	
Sleep hygiene	−0.28** p < 0.001	0.29** p < 0.001	−0.28** p < 0.001	0.22** p < 0.001	1

*p < 0.05; **p < 0.01. Correlations were assessed using Spearman’s rho. LOC: locus of control.

### Cluster analysis

Cluster comparisons (Table 4) were examined using one-way analysis of variance (ANOVA) to assess differences in psychological well-being, insomnia, sleep locus of control, and sleep hygiene across the identified clusters. Where the assumption of homogeneity of variances was violated, Welch’s ANOVA was applied to ensure the robustness of group comparisons.

**Table 4:** Comparison of psychological well-being, insomnia, sleep locus of control, and sleep hygiene across identified clusters.

Variable	Chance dominant (*n* = 215, 51.4%)	Insomnia prone (*n* = 89, 21.3%)	Adaptive (*n* = 114, 27.3%)	F	*p* value
Well-being	72.35 ± 17.86	63.24 ± 21.20	80.98 ± 17.53	21.2	<0.001
Insomnia	3.07 ± 2.25	10.84 ± 3.73	2.61 ± 2.36	183.1	<0.001
Sleep LOC (internal)	20.80 ± 3.23	23.22 ± 3.12	27.82 ± 2.31	261.4	<0.001
Sleep LOC (Chance)	10.22 ± 2.49	7.39 ± 2.08	4.46 ± 1.61	321.8	<0.001
Sleep hygiene	14.19 ± 7.47	18.84 ± 8.03	8.24 ± 7.07	51.8	<0.001

Cluster analysis identified three distinct groups (Table 4; Supplementary Figure S1): Chance Dominant Sleep Profile (51.4%), Insomnia-Prone Sleep Profile (21.3%), and Adaptive Sleep Profile (27.3%). The Insomnia-Prone Sleep Profile was characterized by the lowest well-being scores, highest insomnia severity, poor sleep hygiene, and less favorable beliefs regarding sleep control. Conversely, the Adaptive Sleep Profile exhibited the highest well-being, lowest insomnia severity, optimal sleep hygiene, highest internal sleep locus of control, and lowest chance sleep locus of control. The Chance Dominant Sleep Profile displayed moderate well-being and intermediate levels across sleep-related variables. Significant differences were found among the clusters for all variables (*p* < 0.001).

Each cluster was named accordingly based on their profile across the Sleep Locus of control subscale, insomnia severity and well being. The Chance Dominant profile showed lowest internal SLOC score(20.80, S. D. 2.49), among the three clusters. The Insomnia Prone cluster was named so as they had the highest Insomnia score (10.84, S. D. 3.73) among the other clusters. The Adaptive Profile had the most favorable profile across all measures including lowest insomnia, highest well-being, strongest internal SLOC and optimal sleep hygiene (Table 5).

**Table 5:** Pairwise *post-hoc* comparisons of well-being, insomnia, sleep locus of control, and sleep hygiene across the three clusters.

Variable	Comparison	Mean difference	95% CI	*p*-value	Interpretation
Well-being	C1 vs. C2	2.27	0.77 to 3.79	0.001	Significant
	C1 vs. C3	−2.15	−3.36 to −0.95	<0.001	Significant
	C2 vs. C3	−4.43	−6.08 to −2.79	0.001	Significant
Insomnia	C1 vs. C2	−7.76	−8.78 to −6.76	<0.001	Significant
	C1 vs. C3	0.46	−0.17 to 1.10	0.19	Not Significant
	C2 vs. C3	8.23	7.16 to 9.31	<0.001	Significant
Internal sleep LOC	C1 vs. C2	−2.42	−3.35 to −1.48	<0.001	Significant
	C1 vs. C3	−7.01	−7.73 to −6.28	<0.001	Significant
	C2 vs. C3	−4.59	−5.52 to −3.65	<0.001	Significant
Chance sleep LOC	C1 vs. C2	2.82	2.16 to 3.48	<0.001	Significant
	C1 vs. C3	5.75	5.21 to 6.28	<0.001	Significant
	C2 vs. C3	−2.92	−3.55 to −2.29	<0.001	Significant
Sleep hygiene	C1 vs. C2	−4.65	−7.00 to −2.31	<0.001	Significant
	C1 vs. C3	5.94	3.98 to 7.92	<0.001	Significant
	C2 vs. C3	10.60	8.06 to 13.15	<0.001	Significant

C1, Cluster 1 (Chance Dominant); C2, Cluster 2 (Insomnia prone); C3, Cluster 3 (Adaptive); Levene’s test indicated that the assumption of homogeneity of variance was violated for well-being, insomnia, and sleep hygiene (*p* < 0.05), but met for sleep locus of control (*p* = 0.602). Therefore, Games-Howell test was used for *post-hoc* analysis.

*Post-hoc* pairwise comparisons using the Games–Howell test demonstrated significant differences between the identified clusters across most study variables. For psychological well-being, all pairwise comparisons were significant (*p* ≤ 0.001), with the Adaptive Sleep Profile (C3) reporting the highest well-being scores and the Insomnia-Prone Sleep Profile (C2) reporting the lowest. For insomnia, the Insomnia-Prone Sleep Profile differed significantly from both the Chance Dominant Sleep Profile (C1) and the Adaptive Sleep Profile (C3; *p* < 0.001), whereas no significant difference was observed between C1 and C3 (*p* = 0.19), indicating similarly low insomnia levels in these two groups. Significant differences were observed among all three clusters for both internal and chance sleep locus of control (*p* < 0.001), with the Adaptive Sleep Profile exhibiting the highest internal locus of control and lowest chance locus of control. Similarly, all pairwise comparisons for sleep hygiene were statistically significant (*p* < 0.001), with the Insomnia-Prone Sleep Profile showing the poorest sleep hygiene and the Adaptive Sleep Profile demonstrating the most favorable sleep hygiene practices. These findings support the distinctiveness of the identified sleep-related profiles and their associations with psychological well-being.

### Sleep related variables as predictors of psychological well-being

Multiple linear regression analysis (Table 6) was conducted to examine whether insomnia, sleep locus of control, and sleep hygiene predicted psychological well-being among nursing students. Prior to model estimation, regression assumptions were evaluated. The Breusch–Pagan test indicated no evidence of heteroscedasticity (BP = 5.274, df = 6, *p* = 0.509). Multicollinearity was not a concern, with VIF values ranging from 1.02 to 1.86 and tolerance values ranging from 0.537 to 0.977. Although minor deviations from normality of residuals were observed, the large sample size (*N* = 418) supports the robustness of linear regression estimates to modest departures from normality. Therefore, no data transformations or robust regression procedures were deemed necessary.

**Table 6:** Multiple linear regression analysis of sleep-related variables as predictors of psychological well-being among nursing students.

Predictor	Unstandardized(B)	SE	95% for B	CI	Standardized (β)	95% for *β*	CI	t	*p*
Insomnia	−0.36	0.05	−0.47 −0.26	to	−0.31	−0.40 −0.22	to	−6.76	< 0.001
Internal sleep LOC	0.07	0.07	−0.06 0.21	to	0.06	−0.05 0.18	to	1.14	0.25
Chance sleep LOC	−0.18	0.08	−0.36 −0.01	to	−0.12	−0.24 −0.01	to	−2.02	0.04
Sleep hygiene	−0.08	0.02	−0.14 −0.03	to	−0.13	−0.24 −0.05	to	−2.86	0.004
Age	0.11	0.08	−0.04 0.27	to	0.06	−0.02 0.15	to	1.39	0.16
Gender	−0.81	0.68	−2.17 0.53	to	0.16	−0.44 0.01	to	−1.18	0.23

B, unstandardized regression coefficient; β, standardized regression coefficient; CI, confidence interval; LOC, locus of control. Significant predictors of well-being included insomnia, chance sleep locus of control, and sleep hygiene.

The overall regression model was statistically significant, *F*(6, 411) = 17.14, *p* < 0.001, explaining 20% of the variance in well-being scores (R^2^ = 0.20; Adjusted R^2^ = 0.18). Insomnia was a significant negative predictor of well-being (B = −0.36, *p* < 0.001), followed by chance sleep locus of control (B = −0.18, *p* = 0.04) and sleep hygiene (B = −0.08, *p* = 0.004). Specifically, for every one-unit increase in insomnia score, psychological well-being decreased by 0.36 points. Similarly, for every one-unit increase in chance sleep locus of control and sleep hygiene scores, psychological well-being decreased by 0.18 and 0.08 points, respectively. These findings indicate that greater insomnia severity, stronger beliefs that sleep outcomes are determined by chance, and poorer sleep hygiene practices were associated with lower levels of psychological well-being. Internal sleep locus of control was not a significant predictor of well-being (B = 0.07, *p* = 0.25). Among the predictors, insomnia showed the strongest effect (*β* = −0.31), followed by sleep hygiene (*β* = −0.13) and chance sleep locus of control (*β* = −0.12).

## Discussion

The present study found that nursing students demonstrated moderate psychological well-being, relatively low insomnia severity, and favorable sleep hygiene practices. Cluster analysis identified three distinct sleep-related profiles: insomnia-prone sleep profile, chance-dominant Sleep Profile, and Adaptive Sleep Profile. Students in the Insomnia-Prone Sleep Profile exhibited the poorest psychological well-being, highest insomnia severity, poorer sleep hygiene, and less favorable sleep control beliefs, whereas those in the Adaptive Sleep Profile demonstrated the most favorable psychosocial and sleep-related characteristics. Regression analysis further revealed that higher insomnia severity, poorer sleep hygiene, and stronger beliefs that sleep outcomes are due to chance were significantly associated with lower psychological well-being, with insomnia having the most important association. These factors collectively explained a low, however meaningful, proportion of the variation (20%) in psychological well-being among nursing students.

These findings are partially consistent with previous international studies reporting that although nursing students commonly experience sleep disturbances, variations in sleep behaviors and coping mechanisms may influence psychological outcomes, including well-being (Ravi and Mohamed, 2022; Kundayi Ravi and Mohamed, 2024; Mohamud et al., 2025; Mithurja et al., 2026). A study conducted among Spanish nursing students reported that poor sleep habits and short sleep duration were prevalent and negatively affected academic functioning and health outcomes (Gallego-Gómez et al., 2021). The comparatively moderate well-being observed in the present study may be related to adaptive coping strategies, social support, and resilience among students despite the academic and clinical stressors (Naz et al., 2024; Loureiro et al., 2025).

A notable finding of the present study was the identification of three distinct sleep-related profiles in nursing students. Nearly half of the participants fell into the Chance Dominant Sleep Profile, representing a transitional group with moderate sleep functioning that still requires attention and preventive strategies. Nearly one-fifth of the students belonged to the Adaptive Sleep Profile, characterized by the most favorable psychosocial and sleep-related attributes. The remaining portion, over a quarter, fell into the Insomnia-Prone Sleep Profile, which was associated with markedly poorer psychological well-being, greater insomnia severity, poorer sleep hygiene, and less adaptive sleep control beliefs. These findings suggest a clear continuum of sleep health ranging from maladaptive to adaptive functioning, while also highlighting that a substantial segment of the population experiences suboptimal sleep-related functioning. This person-centered pattern is consistent with recent evidence suggesting that sleep health among nursing and university students is multidimensional rather than uniform. Similar latent profile research among nursing interns has also reported distinct circadian and sleep typologies characterized by adaptive and maladaptive subgroups, with maladaptive profiles showing poorer psychological functioning and higher perceived stress (Wu R. et al., 2022). Likewise, studies among university populations have demonstrated that poor sleep quality and dysfunctional sleep behaviors cluster together and are strongly associated with lower psychological well-being and increased distress, supporting the pattern observed in the Insomnia-Prone Sleep Profile in the present study (Belingheri et al., 2022). The finding that only a small proportion of students belonged to the Adaptive Sleep Profile, while the majority fell into the Chance-Dominant or Insomnia-Prone groups, is consistent with recent reports from nursing student populations showing a high prevalence of poor sleep quality and irregular sleep–wake patterns, particularly in clinical training environments (Kayaba et al., 2021).

The current findings can also be viewed through the lens of the Cognitive Behavioral Theory (CBT) of insomnia. This theory suggests that insomnia persists not only due to physiological arousal but also because of maladaptive sleep-related thoughts, dysfunctional beliefs, and harmful behavioral patterns (Tang et al., 2023). Factors such as poor sleep hygiene, irregular sleep routines, excessive screen time, and negative expectations about sleep create a cycle of sleep problems and psychological distress. Students with more severe insomnia might experience increased worry about sleep, higher emotional arousal, and decreased coping abilities, which can harm their psychological well-being (Nielson et al., 2023; Tang et al., 2023). The link between insomnia and lower psychological health observed in this study aligns with cognitive-behavioral models that highlight the bidirectional relationship between sleep issues and emotional health (Ravi and Mohamed, 2022; Ravi and Mohamed, 2022; Tang et al., 2023; Amalia and Asih, 2024).

The statistically significant association observed between insomnia severity, poor sleep hygiene, strong beliefs that sleep is determined by chance, and lower psychological well-being indicates the important role these sleep-related factors play in influencing well-being among university students (Igbokwe, 2021; Gardani et al., 2022). These findings are consistent with previous international and Indian studies reporting that insomnia and unhealthy sleep behaviors are significantly associated with psychological distress, stress, anxiety, depression, and impaired academic functioning in students (Ghrouz et al., 2021; Carrión-Pantoja et al., 2022; Alwhaibi and Al Aloola, 2023; Peng et al., 2025; Sleiay et al., 2026). Poor sleep hygiene may be associated with inadequate sleep duration, irregular sleep patterns, and daytime fatigue, which can negatively affect emotional regulation and psychological well-being. Similarly, students with stronger chance-related beliefs about sleep may perceive less personal control over their sleep outcomes, which may be associated with feelings of helplessness and poor psychological adjustment (Vieira et al., 2023). In contrast, the internal sleep locus of control did not significantly predict psychological well-being after adjusting for other variables. A possible explanation may be that although students with stronger internal control beliefs may attempt to regulate their sleep behaviors, external academic demands, clinical schedules, stress, and environmental factors may limit their ability to maintain healthy patterns of sleep (Vieira et al., 2023). Additionally, cultural and educational factors specific to Indian nursing education, including intensive academic expectations and hostel-based living environments, may influence sleep behaviors, irrespective of personal control beliefs (Ravi et al., 2025; Kundayi Ravi et al., 2026).

The regression analysis in the present study, the sleep related varaibles accounted for only 20% of variance in the psychological well-being (Adjusted R20.18). This indicates that 80% of variance in psychological well-being is not explained by insomnia, sleep locus of control or sleep hygiene. Hence it can be understood that psychological well-being in nursing students is influenced by broader concepts outside sleep which included, but not limited to, resilience, perceived social support, self esteem, personality traits and other contextual factors (Ravi and Mohamed, 2022; Shdaifat et al., 2024; Kundayi Ravi et al., 2026).

From a health behavior standpoint, these results highlight the importance of comprehensive strategies that target both behavioral and cognitive factors influencing sleep duration. Initiatives focused on enhancing sleep hygiene, boosting self-regulation skills, and increasing personal control over sleep may foster better psychological well-being among nursing students and they need to be tested using inetreventional research. Since sleep issues can affect future clinical skills, professional performance, and patient care, nursing programs should consider adding sleep health promotion efforts to student support and curricula.

### Implications for the study

This study has important implications for improving the sleep health and psychological well-being of nursing students. Identifying distinct psychosocial sleep-related profiles-vulnerable (Insomnia Prone), resilient (Adaptive), and intermediate (Chance Dominant)–highlights the heterogeneity within this population and underscores the need for tailored interventions. In students with Insomnia Prone profile, cognitive behavioral therapy can be beneficial to address maladaptive behaviors. Interventions incorporating cognitive restricting can help students wiyh chance dominant profile to shif their locus of control more internally. Furthermore, the findings emphasize the importance of addressing sleep-related issues within nursing education programs by providing structured sleep hygiene and stress management psychoeducation regardless of the cluster they belong to.

### Strengths

The study employed validated and reliable instruments to robustly measure sleep and well-being variables, while the use of two-stage cluster analysis combined with regression modeling provided comprehensive insights into the multifaceted nature of sleep and mental health among nursing students, a population vulnerable to academic and clinical demands. By addressing an underexplored population and integrating cognitive (SLOC) and behavioral (sleep hygiene, insomnia) factors, this study contributes novel findings relevant to both regional and global literature. A key strength of this study is the identification of three empirically derived, clinically distinct sleep profiles among university students, which translates complex, multidimensional sleep data into a practically usable framework. This can enable matching intervention strategies to student profiles rather than treating sleep difficulties and psychological well-being as uniform constructs across the entire student population.

### Limitations and recommendations for future research

The cross-sectional design of this study limits causal inferences, highlighting the need for longitudinal research to clarify the temporal relationships among insomnia, sleep hygiene, sleep locus of control (SLOC), and psychological well-being. The tools sused in the study, though found to have good reliability, have not been validated in Indian population. This should be considered while interpreting results. Convenience sampling from two nursing colleges in the Northwestern region of India introduces potential selection bias, restricting the generalizability of the findings beyond the participating institutions. Additionally, reliance on self-reported data may be affected by recall bias or social desirability, particularly concerning sleep behaviors and psychological well-being. As all the variables under study were collected only through self- reported questionnaire, common method bias cannot be ruled out and could have resulted in inflated association between variables. Objective measures of sleep, including actigraphy and polysomnography could have been used rather than relying on subjective reports of sleep related variables. Factors like depression and anxiety wer enot measured in this study; their loinkage with psychological weel being and sleep is hence not accounted for in this study. Furthermore, sleep is a dynamic variable, and the factors measured in this study could fluctuate across academic sessions and seasons of the year. Hence, the stability of the identified clusters may be considered situational rather than dispositional.

Future research should employ randomized sampling across diverse nursing institutions to enhance external validity and incorporate objective sleep assessments such as actigraphy or polysomnography alongside self-reports. The regression model in this study did not account for key variables that could influence psychological well-being. Uncontrolled confounding factors such as academic stress, clinical workload, and lifestyle variables may also influence sleep and well-being outcomes Future studies should account for these variables such as resilience, socioeconomic status, anxiety traits, physical activity, screen usage and residence type to isolate independent effect of sleep related factors on psychological well-being. Investigating tailored interventions targeting maladaptive sleep beliefs and behaviors within the identified clusters could help evaluate their efficacy in improving well-being. Furthermore, exploring additional psychosocial and environmental mediators or moderators, including stress, coping, and social support, as well as cultural and contextual influences on SLOC and sleep hygiene practices among nursing students across different regions, will deepen the understanding of the sleep-well-being relationship.

## Conclusion

This study underscores the complex interplay between insomnia severity, sleep hygiene behaviors, and sleep locus of control in shaping the psychological well-being of nursing students. The identification of varying profiles highlights the need for targeted, multifaceted interventions that address both the behavioral and cognitive aspects of sleep health. Sleep hygiene, insomnia severity, and maladaptive sleep control beliefs are linked to mental health outcomes in this vulnerable population. Hence support is needed in these sleep related beahviours so that students are wellsupported in their academic performance and future clinical competence.

## Data Availability

The raw data supporting the conclusions of this article will be made available by the authors, without undue reservation.
