# Supplementary material for: Profiles of sleep hygiene, insomnia, sleep locus of control, and psychological well-being among nursing students: a cluster analysis
Source: Front Psychol. 2026 Sep 3;17:1920757. doi: 10.3389/fpsyg.2026.1920757 (PMC13582283; doi:10.3389/fpsyg.2026.1920757)
Supplement: Supplementary file 1 [file Supplementary_file_1.docx]

**Supplementary file 1: Data collection instrument**

**SECTION 1:Profile of participants**

1. How old are you
2. What is your gender?
   a. Male
   b. female
3. In which program are you currently enrolled in?
   a. BSN
   b. msn
   c. Diploma
4. In which year are you studying?
   a. 1
   b. 2
   c. 3
   d. 4
5. Do you have any chronic illness?
   a. Yes
   b. No

**Section 2: Athens Insomnia Scale**

| 1. SLEEP INDUCTION (time it takes you to fall asleep after turning-off the lights) | No problem | Slightly delayed | Markedly delayed | Very delayed or did not sleep at all |
| --- | --- | --- | --- | --- |
| AWAKENINGS DURING THE NIGHT | No problem | Minor problem | Considerable problem | Serious problem or did not  sleep at all |
| FINAL AWAKENING EARLIER THAN DESIRED | Not earlier | A little earlier | Markedly earlier | Much earlier or did not sleep at all |
| TOTAL SLEEP DURATION | Sufficient | Slightly insufficient | Markedly insufficient | Very insufficient or did not sleep at all |
| OVERALL QUALITY OF SLEEP (no matter how long you slept) | Satisfactory | Slightly unsatisfactory | Markedly unsatisfactory | Very unsatisfactory or did not sleep at all |
| SENSE OF WELL-BEING DURING THE DAY | Normal | Slightly decreased | Markedly decreased | Very decreased |
| FUNCTIONING (PHYSICAL AND MENTAL) DURING THE DAY | Normal | Slightly decreased | Markedly decreased | Very decreased |
| SLEEPINESS DURING THE DAY | None | Mild | Considerable | Intense |

#### **Section 3:** Sleep hygiene index

|  | Never | Rarely | Sometimes | Frequently | Always |
| --- | --- | --- | --- | --- | --- |
| I take daytime naps lasting two or more hours. |  |  |  |  |  |
| I go to bed at different times from day to day. |  |  |  |  |  |
| I get out of bed at different times from day to day. |  |  |  |  |  |
| I exercise to the point of sweating within 1 hr of going to bed |  |  |  |  |  |
| I stay in bed longer than I should two or three times a week |  |  |  |  |  |
| I use alcohol, tobacco, or caffeine within 4hrs of going to bed or after going to bed. |  |  |  |  |  |
| I do something that may wake me up before bedtime (for example: play video games, use the internet, or clean). |  |  |  |  |  |
| I go to bed feeling stressed, angry, upset, or nervous |  |  |  |  |  |
| I use my bed for things other than sleeping or sex (for example: watch television, read, eat, or study) |  |  |  |  |  |
| I sleep on an uncomfortable bed (for example: poor mattress or pillow, too much or not enough blankets) |  |  |  |  |  |
| I sleep in an uncomfortable bedroom (for example: too bright, too stuffy, too hot, too cold, or too noisy). |  |  |  |  |  |
| I do important work before bedtime (for example: pay bills, schedule, or study) |  |  |  |  |  |
| I think, plan, or worry when I am in bed. |  |  |  |  |  |

**Section 4: Sleep locus of control scale**

|  | agree very Strongly | agree Strongly | agree | Disagree | Strongly Disagree | Very Strongly Disagree |
| --- | --- | --- | --- | --- | --- | --- |
|  |  |  |  |  |  |  |
| If I take care of myself , I can avoid insomnia |  |  |  |  |  |  |
| Whether I have insomnia is entirely up to me |  |  |  |  |  |  |
| Good sleep is largely as a matter of luck |  |  |  |  |  |  |
| No matter what I do ,if I am going to have a sleepless night , I will |  |  |  |  |  |  |
| When I have insomnia ,I know it is because of something I have done(not having enough time to relax, worrying bout the things that I cant control, worrying about not sleeping) |  |  |  |  |  |  |
| People who never get insomnia are just plain lucky |  |  |  |  |  |  |
| Peoples insomnia results from their won carelessness |  |  |  |  |  |  |
| I am directly responsible for my sleep |  |  |  |  |  |  |

**Section 5 : sleep hygiene**

**WHO 5 item well being**

|  | **All of the time** | **Most**  **of the time** | **More**  **than half the time** | **Less**  **than half the time** | **Some**  **of the time** | **At no time** |
| --- | --- | --- | --- | --- | --- | --- |
| I have felt cheerful in good spirits. |  |  |  |  |  |  |
| I have felt calm and relaxed. |  |  |  |  |  |  |
| I have felt active and vigorous. |  |  |  |  |  |  |
| I woke up feeling fresh and rested. |  |  |  |  |  |  |
| My daily life has been filled with  things that interest me. |  |  |  |  |  |  |
