# Supplementary material for: Profiles of sleep hygiene, insomnia, sleep locus of control, and psychological well-being among nursing students: a cluster analysis
Source: Front Psychol. 2026 Sep 3;17:1920757. doi: 10.3389/fpsyg.2026.1920757 (PMC13582283; doi:10.3389/fpsyg.2026.1920757)
Supplement: Supplementary file 2 [file Supplementary_file_2.docx]

Standardised Z score

|  | Insomnia | Internal Sleep LOC | Chance sleep LOC | Sleep Hygiene |
| --- | --- | --- | --- | --- |
| 1 | -0.363 | -0.57 | -0.66 | 0.07 |
| 2 | 1.48 | -0.002 | 0.19 | 0.63 |
| 3 | -0.47 | 1.09 | 1.08 | -0.63 |

**Figure S1: Cluster Profiles of Sleep-Related Factors and Psychological Well-Being Among Nursing Students**
